# Reconstructing self from the illness: a constructivist grounded theory study of posttraumatic growth in patients with Crohn’s disease

**DOI:** 10.1186/s12876-023-02878-1

**Published:** 2023-07-18

**Authors:** Ying Wang, Chen Zhang, Yunxian Zhou

**Affiliations:** School of Nursing, Zhejiang Chinese Medical University, 548 Binwen Road, Binjiang District, Hangzhou, 310053 Zhejiang China

**Keywords:** Crohn’s disease, Inflammatory bowel disease, Posttraumatic growth, Qualitative research, Grounded theory

## Abstract

**Background:**

Some patients with Crohn’s disease report posttraumatic growth, which can promote reductions in anxiety and depression, and improve the patient’s quality of life. However, the process of posttraumatic growth remains unclear. The objectives of this study were to explore the social-psychological process of posttraumatic growth in patients with Crohn’s disease in the context of Chinese culture and construct an interpretive understanding based on the perspectives of patients.

**Methods:**

This research adopted Charmaz’s constructivist grounded theory. Nineteen participants with Crohn’s disease were selected by purposive and theoretical sampling from three hospitals in Hangzhou, Zhejiang Province, as well as from the China Crohn’s & Colitis Foundation. In-depth interviews were conducted. Data analysis was based on initial, focused and theoretical coding strategies, and methods such as constant comparison and memo writing were adopted. The Consolidated Criteria for Reporting Qualitative Research, a 32-item checklist for interviews and focus groups, was utilised.

**Results:**

An interpretive understanding of posttraumatic growth in patients with Crohn’s disease was constructed. The core category was “reconstructing self from the illness”, which included the following four categories: “suffering from the illness”, “accepting the illness”, “dancing with the illness” and “enriching life beyond the illness”. However, not every patient reached the last phase. Before patients enter the next stage, they might begin a new cycle by experiencing new suffering or discovering new unacceptable facts about the illness.

**Conclusions:**

This interpretive understanding reveals a growth process centred on “reconstructing self from the illness”. These findings provide knowledge on the process of posttraumatic growth in patients with Crohn’s disease within the wider sociocultural context.

## Background

Crohn’s disease, a form of inflammatory bowel disease, is a chronic inflammatory disease of the gastrointestinal tract; its pathogenesis is not well understood [1]. The peak incidence of Crohn’s disease is between 18 and 35 years of age [1]. A retrospective study estimated that the incidence of Crohn’s disease in Guangzhou, China is approximately 1.36/100,000, and this number is expected to increase in the future [2]. The main clinical manifestations of Crohn’s disease are diarrhoea, abdominal pain and haematochezia [1]. Compared with ulcerative colitis, Crohn’s disease can involve the whole gastrointestinal tract and Crohn’s patients are prone to extraintestinal manifestations such as erythema nodosum and complications such as bowel obstruction [3]. Therapies for Crohn’s disease, such as hormonotherapy, may cause intolerable side effects and complications. At present, there is no cure for Crohn’s disease and relapse after remission is common [1], which exacerbates patient suffering. Meanwhile, because of the need for surgery or biotherapy, Chinese patients with Crohn’s disease are likely to experience a heavy economic burden. A previous study [4] revealed that the mean annual direct cost per Chinese patient with inflammatory bowel disease is approximately US $11,668.68, and nearly 98% of Crohn’s patients are worried about their financial situation.

In addition to the physical suffering and heavy economic burden, Crohn’s disease also negatively impacts a patient’s mental health. Physical changes that patients experience due to treatment, such as weight loss and colostomy, increase the risk of stigma. Patients also often experience psychological distress, including embarrassment, anxiety and depression [5–8]. These negative psychological experiences can aggravate the patient’s condition [5], increase the hospitalization and relapse rates, affect the treatment effect [9] and lead to a decrease in the patient’s quality of life and treatment compliance [10, 11].

In reviewing empirical studies and related theories, it was found that posttraumatic growth can improve the quality of life of traumatized individuals, promote their social adaptation, and improve their mental health [12–16]. The term posttraumatic growth was first coined by Tedeschi and Calhoun [17]. Different theorists have proposed diverse models of posttraumatic growth based on Western culture [18, 19]. Posttraumatic growth is initiated by an encounter with highly challenging circumstances and is prompted by a challenge to one’s assumptive world or core beliefs [19–21]. It can be understood as a cluster of positive changes resulting from a complex combination of cognitive, emotional and social processes [19–21].

In the field of medical care, posttraumatic growth is not just experienced among patients facing an acute trauma or life-threatening disease, but also among individuals living with a chronic disease [22, 23]. Lyon et al. [24] explored the process by which posttraumatic growth developed following acquired brain injury in 10 participants. Four inter-connected themes were constructed: “living with a life-changing injury”, “trying to ‘beat it’ and acceptance”, “identifying with a new you and others” and “meaningful positive change” [24]. Zhai et al. [25] developed a posttraumatic growth theory among 24 Chinese women with breast cancer. The basic social process identified was “emerging from the ‘ku’”, which comprised four categories: “confronting challenges”, “orienting to reality”, “accommodating the illness” and “transforming their lives” [25] .

Several studies within the field of inflammatory bowel disease have also focused on posttraumatic growth. A Canadian study [26] explored the positive changes experienced by patients with inflammatory bowel disease after diagnosis and generated the following five themes based on the findings: interpersonal relations, personal growth, valuing life, new life paths and spiritual growth. Similarly, studies in China [27, 28] have found that some patients with Crohn’s disease undergo posttraumatic growth, including the formation of stronger and closer relationships with others, living in the present, and reordering priorities. Together, the above research indicates that some patients with Crohn’s disease experience posttraumatic growth. However, few published studies [22, 29, 30] have focused on the process of posttraumatic growth in these patients. The insight from breast cancer [25] may not be applicable to the Crohn’s disease population given the difference in gender and age. Most of the posttraumatic growth models have been developed and examined from a Western perspective. However, there are important differences between Eastern and Western cultures. For example, Western culture emphasizes self-control and personal determinism, while Eastern culture is based on a fatalistic or deterministic framework [31, 32]. Individualistic and collectivistic cultures may also affect an individual’s coping and response to trauma [28, 31]. Given that cultural elements play an important role in the behaviour of individuals in the aftermath of trauma, the applicability of a Western-based posttraumatic growth theory to explain the experience of Chinese Crohn’s disease patients is yet to be determined. Therefore, the objective of this study was to explore the psychosocial process of posttraumatic growth in Chinese patients with Crohn’s disease and construct an interpretive understanding in the context of Chinese culture.

## Methods

### Design

This research adopted Charmaz’s constructivist grounded theory. Constructivist grounded theory assumes multiple realities and mutual construction by researchers and participants; the construction process is thought to be affected by the time, place and environment [33]. Symbolic interactionism places a clear emphasis on meaning, interpretation, self and social interaction [34, 35]. The constructivist view fits well with the premise of symbolic interactionism. Thus, in interpreting the posttraumatic growth of patients with Crohn’s disease, the focus of this study was on human action and interaction as dynamic processes, wherein situations were defined and meanings were constructed. Accordingly, constructivist grounded theory was an ideal approach to achieve the research objective.

### Participants and sampling

The inclusion criteria were as follows: (1) diagnosis of Crohn’s disease according to the Chinese criteria released in 2018 [1], (2) 18 years of age or older and willing to share their illness experience, and (3) experienced a self-reported positive change following Crohn’s disease diagnosis. Patients who had other life-threatening diseases or mental diseases were excluded.

Initially, purposive sampling was employed. Patients who met the above inclusion criteria were identified by CZ, a female postgraduate nursing student who was engaged in clinical practice at three tertiary hospitals and was volunteering at the China Crohn’s & Colitis Foundation in Hangzhou, Zhejiang Province. During the sampling process, the researchers approached eligible patients, explained the reasons for conducting the research, and invited them to participate in an interview conducted face-to-face or online. To recruit more participants, a snowball sampling strategy was later adopted. During the gradual development of the theoretical interpretation, theoretical sampling was employed [33]. For example, when the subcategory “exploring joys of life” was being developed, patients who reported a variety of ways in which their lives were enriched were selected to provide further insight into this subcategory. Sampling ceased when theoretical saturation was achieved. In total, 21 patients with Crohn’s disease were invited to participate in the interviews, and 19 patients agreed to participate; two patients declined because of physical discomfort. The sociodemographic and clinical characteristics of the participants are presented in Table 1.

**Table 1:** Study participants’ socio-demographic and clinical characteristics

ID	Age	Gender	Place of residence	Marital status	Education level	Occupation	Religion status	Per capita monthly household income(Yuan)	Number of surgeries	Harvey-Bradshaw simple index^a^	Course of disease (months)
1	25	Female	Zhejiang	Single	Tertiary or above	Clerk	No religion	3000–5000	0	0	8
2	20	Male	Zhejiang	Single	Tertiary or above	Student	No religion	5001–10,000	1	6	36
3	23	Male	Zhejiang	Single	Tertiary or above	Student	No religion	3000–5000	1	0	129
4	34	Male	Zhejiang	Single	Tertiary or above	Clerk	No religion	5001–10,000	0	2	101
5	45	Female	Guangdong	Single	Tertiary or above	Freelance	Buddhist	3000–5000	0	1	240
6	24	Male	Shanghai	Single	Tertiary or above	Student	No religion	> 10,000	0	0	36
7	27	Male	Shanghai	Single	Tertiary or above	Clerk	No religion	5001–10,000	1	5	82
8	39	Male	Jiangsu	Married	Tertiary or above	Engineer	No religion	> 10,000	0	1	18
9	34	Female	Zhejiang	Married	Tertiary or above	Freelance	Buddhist	3000–5000	1	0	84
10	25	Male	Jiangsu	Single	Tertiary or above	Unemployed	No religion	> 10,000	0	1	73
11	32	Male	Jiangsu	Married	Tertiary or above	Engineer	Christian	> 10,000	0	0	120
12	35	Male	Jiangsu	Married	Tertiary or above	Engineer	No religion	> 10,000	0	0	74
13	31	Male	Chongqing	Single	Tertiary or above	Freelance	No religion	> 10,000	1	3	20
14	44	Female	Zhejiang	Married	Tertiary or above	Teacher	Buddhist	5001–10,000	1	2	80
15	19	Female	Anhui	Single	Tertiary or above	Student	No religion	3000–5000	3	0	72
16	47	Male	Zhejiang	Married	Secondary	Freelance	No religion	> 10,000	1	1	132
17	39	Female	Zhejiang	Married	Tertiary or above	Self-employed entrepreneur	No religion	> 10,000	0	0	84
18	50	Female	Zhejiang	Married	Tertiary or above	Teacher	No religion	5001–10,000	1	5	144
19	28	Female	Zhejiang	Single	Tertiary or above	Clerk	No religion	3000–5000	4	6	109

^a^ Disease activity indices were assessed using the Harvey-Bradshaw simple index [36], with a total score of 19. Harvey-Bradshaw simple index <5 is defined as remission; 5 to 7, as mildly active disease; 8–16, as moderately active disease; and >16, as severely active disease [37]

### Data collection

CZ interacted with most of the recruited patients for six months before the interview to establish rapport. She conducted the in-depth interviews, with the guidance of YZ (a female PhD qualitative expert who works within the School of Nursing), from December 2019 to October 2020. Before the formal interview, the researcher explained the purpose of the research and strategies to protect the patient’s privacy and anonymity. After informed consent was obtained, the researcher began the interview with casual conversation to put the patient at ease. Initially, the scope of the interview questions was relatively broad, such as “What positive changes have you made during your interaction with the disease?“ With the development of the emerging categories, the interview questions became more focused, such as “How did you go from unwillingness to accept to acceptance of the disease”. The researcher encouraged participants to express their views on their own terms.

Face-to-face interviews (n = 6) were conducted in coffee shops or parks. After the outbreak of the COVID-19 pandemic, video (n = 6) and telephone interviews (n = 7) were conducted in quiet and private environments. One of the limitations of telephone interviews is that they do not allow nonverbal and environmental information to be acquired [38, 39]. To minimize the impact of this limitation, researchers paid more attention to auditory cues during the interviews. In this study, each participant was interviewed once; the average duration of the video and telephone interviews was generally longer than that of the other interview forms, and the level of richness was similar.

No one else was present during any of the interviews. All interviews were audio-recorded and lasted 45 to 105 min, with a median length of 75 min. The data were transcribed verbatim by the researchers. Field notes were written within 24 h of the interviews to record details of the observations, interactions, environment and body language of the participants.

### Data analysis

**Fig. 1:**
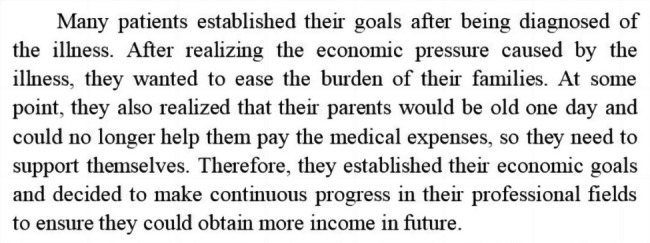
An example of a part of a memo regarding planning for future

Researchers (YW [a female PhD candidate with comprehensive experience in grounded theory], CZ and YZ) used constructivist grounded theory methods to analyse the data. Data analysis and data collection were performed simultaneously and iteratively [33]. The analysis was performed in Chinese, but the codes and quotes were translated into English by the researchers for the purpose of writing this paper. First, initial coding was manually performed to explore the data line-by-line [33]. Special attention was paid to the preservation of the actions and processes. For example, when the participant described, “I tried some sports that I have never done before”, it was coded as “increasing new attempts”. Meanwhile, the researchers asked themselves questions such as, “Why did the patient try to increase new attempts?” Second, focused coding was performed by sifting through the large number of initial codes to form categories and subcategories [33], such as “assuming the worst” and “securing future life”. Third, theoretical coding was performed to write story lines; this was achieved through continuous processing of the categories and the relations between categories to obtain the “core category” [33]. The coding process was nonlinear but cyclic. Regular discussions were undertaken in regard to the adjustment of coding and the meanings of subcategories and categories. Meanwhile, constant comparison and memo writing were employed to improve the quality of the analysis. An example of a memo is provided (Fig. 1). Furthermore, a reflexive journal was kept to record the thoughts, problems and decisions encountered during the analysis. The journal was included as a data source and as a contribution to theoretical sensitivity during the analysis.

### Ethical considerations

#### Ethical approval

for this study was obtained from the Ethics Committee of Zhejiang Chinese Medical University. The participants were informed about the purpose and method of the study, and the researchers obtained written or electronic signed informed consent from all participants. Participants were free to withdraw at any time without penalty. During the interview, the level of comfort of each participant was continuously assessed. Participants were free to terminate the interview or reschedule it for a later time if they experienced discomfort or anxiety. Participants were also provided with a referral to a free counselling service if necessary. All interview data were only used for this research, and only the research team could access these data.

### Rigour

The rigour of the research is reflected in the following aspects: (1) two practice interviews were conducted prior to the formal interviews and the interview guide and data analysis were regularly discussed with qualitative research experts, and (2) the researchers constantly engaged in self-reflection by keeping a reflective journal, which helped them to reduce the influences of their past experiences, such as their own personal posttraumatic growth experiences and assumptions regarding the treatment experiences of the patients.

## Results

An interpretive understanding of posttraumatic growth in patients with Crohn’s disease was constructed in this study. The core category was “reconstructing self from the illness”, which included the following four categories: “suffering from the illness”, “accepting the illness”, “dancing with the illness” and “enriching life beyond the illness”.

### The core category: reconstructing self from the illness

Reconstructing self from the illness is the core category identified in this study. The relapsing and remitting nature of Crohn’s disease is a lifelong threat for patients, and the self is constantly reconstructed during interaction with the disease. In this process, the self’s perception of the disease will change, and the relationship between the self and the disease will also change. The process of self-reconstruction reflects a general trend of growth.

After being diagnosed with Crohn’s disease, patients reported suffering from physical distress and negative emotions. Due to repeated medical treatments, the patients’ normal study and work were seriously affected, resulting in them lagging behind their peers to a certain extent. This left the patients with the impression that the illness is “uncontrollable and scary”. At this stage, the self was shrouded by the disease; this is the “suffering from the illness” phase. The patients’ perceptions of the illness gradually changed to “controllable and not scary” as they discovered that the illness was a reality that they could not change, or as treatment began to work and the disease improved, or as they learned positive information about the illness and felt supported and encouraged by the outside world. The seeds of hope began to grow in their hearts even though they were still suffering from the illness and the illness made them imperfect. The relationship between the self and the illness also changed from the self being completely shrouded by the illness to the self emerging from the shadow of the illness; this is the stage of “accepting the illness”. When the disease entered remission, the patients who relied entirely on hospitals and clinicians rather than self-management were likely to return to their previous lives. Some patients recognised the need to live in harmony with their illness by relying on themselves. They reflected on the past, responded according to the situation, and planned for the future within their abilities. At this stage, the patients treated the illness as a lifelong fellow traveller and began to live in harmony with the illness; this is the “dancing with the illness” stage. As time went by, the patients became increasingly comfortable with the illness, and the self grew further; the illness became less and less threatening to the self until it became a part of the self. The patients hoped to break through the limits of the illness to a certain extent, to no longer be constrained by their ‘patient’ identity, to seek fun and satisfaction, to breakthrough beyond the illness, and to make life more meaningful; this is the “enriching life beyond the illness” stage.

**Fig. 2:**
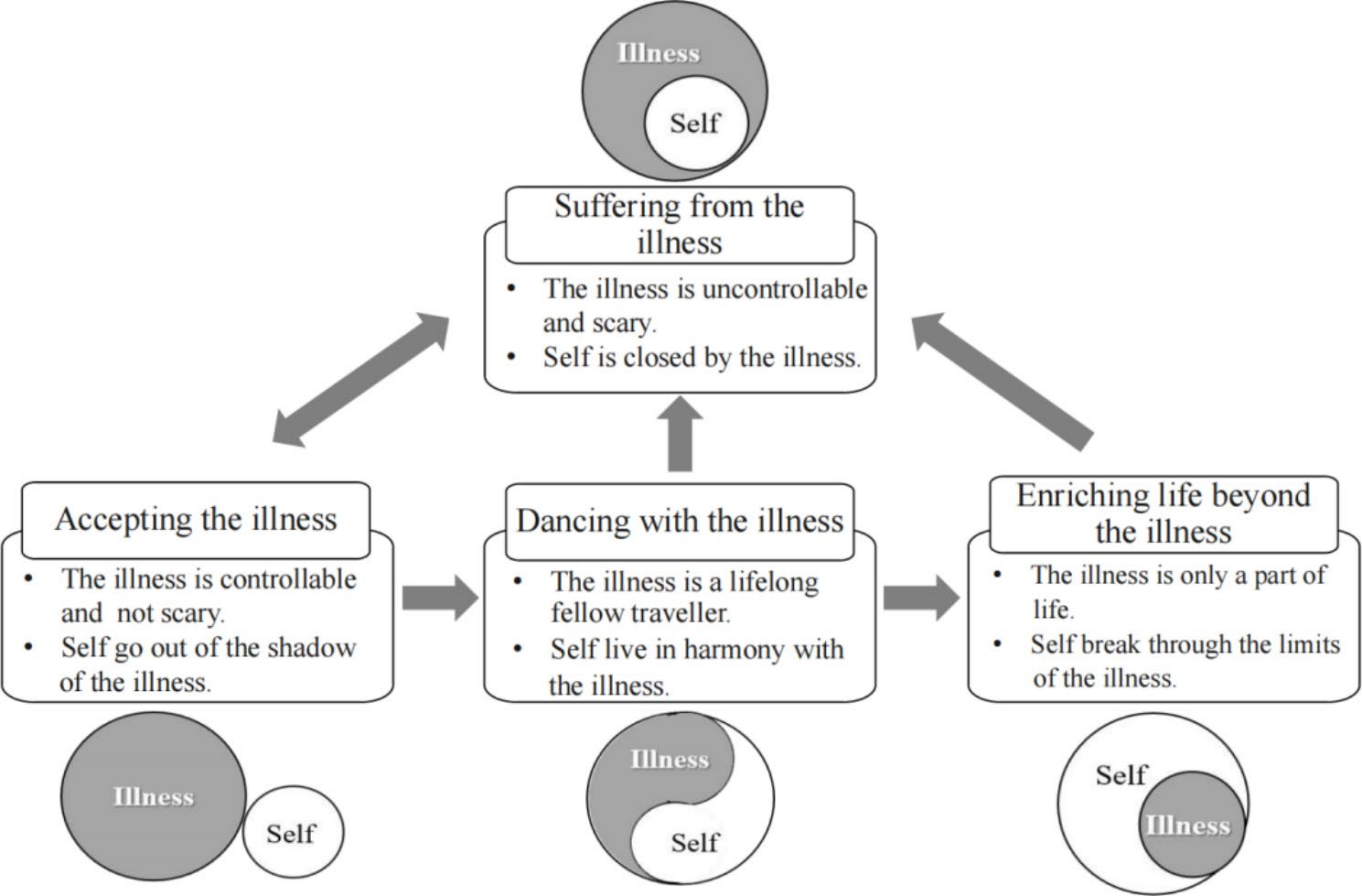
Core category of reconstructing self from the illness

**Fig. 3:**
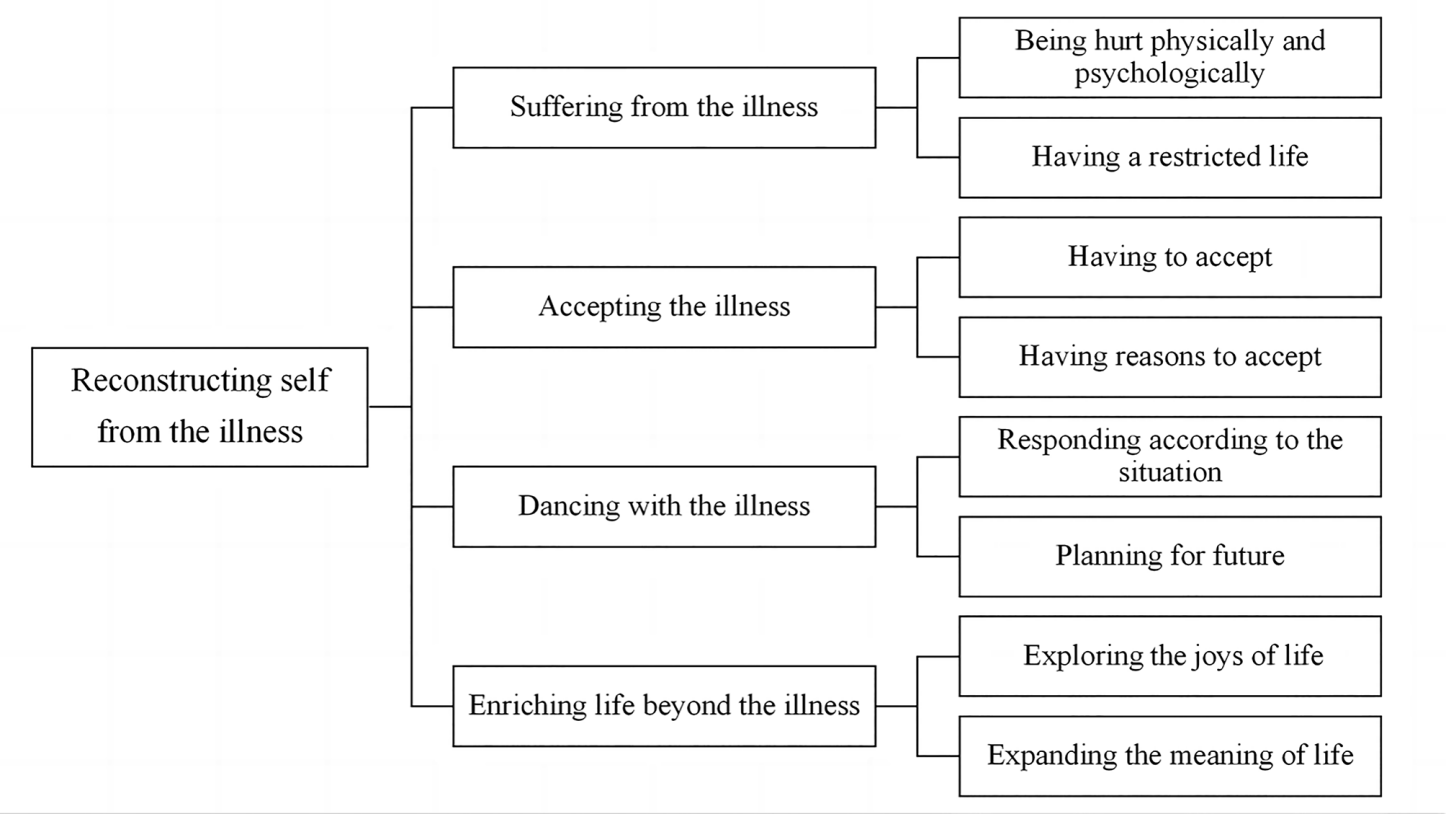
Categories and subcategories of reconstructing self from the illness

The category “suffering from the illness” was the premise underlying the participants’ experience of posttraumatic growth. After experiencing this phase, the participants gradually “accepted the illness”, “danced with the illness” and had an “enriched life beyond the illness”; growth occurred in these three phases. However, not every patient reached the last phase. Moreover, before participants entered the next phase, they might enter a new cycle by experiencing new suffering and discovering new unacceptable facts about the illness. The specific relationship of each phase is presented in Fig. 2. The following discussion provides a depiction of each phase in the form of a category and its subcategories (Fig. 3).

### Suffering from the illness

This category relates to the haunting past experiences that kept intruding into the consciousness of participants which, in turn, seriously affected the participants’ normal lives. At this stage, Crohn’s disease was perceived as uncontrollable and scary for the participants. Some were unable to accept the illness, and many had strong feelings that their self was shrouded and enveloped by the shadow of the illness.

#### Being hurt physically and psychologically

This subcategory relates to the impact of the illness on both the body and mind of the participants. These two impacts often existed at the same time, and changes in one often led to changes in the other.

Before their Crohn’s disease was effectively controlled, the participants suffered from various symptoms, and their daily lives were occupied by the illness. Every day was torture for them.

*I had haematochezia in the morning and couldn’t sleep at night. I suffered every day. (Participant 2)*

The participants also suffered from the side effects of the medication or the pain of their therapies, such as dressing changes after surgery.

*The dressing changes every day was particularly painful. I asked my mother more than once to leave hospital and go home right now. (Participant 15)*

Apart from being tortured physically, patients also experienced various negative emotions. Since Crohn’s disease is not a common illness in China, the first reaction of most participants was fear when being diagnosed. Because they were new to the illness, the information they found on the internet and the information provided by their doctors was sometimes not able to ease their fear and sometimes even deepened it.

*The doctor told me the situation was very severe, and all my colon had to be removed, and then my small intestine was to be directly connected to the rectum, which caused me to feel extremely panicked. (Participant 13)*

Subsequently, due to the uncertainty of the effects of the treatments, participants often felt overwhelmed.

*If I had an operation, my body would suffer greatly first. Second, the operations would cost tens of thousands (Yuan). Third, after the surgeries, I must convalesce at home. Because of these three major problems, I was overwhelmed at that time. (Participant 13)*

As the treatment progressed, some participants faced insufficient treatment effects or a total lack of treatment effect. The participants felt annoyed and anxious due to the constant adjustments to their treatment plans. When the condition persisted for a long time with no improvement, the participants felt desperate. They lost the concepts of “tomorrow”, “future” and “hope”, and some even abandoned “today” and chose to give up on themselves.

*I tried all kinds of medicines, but nothing worked. I was desperate. I felt there was no hope in my life and everything was gloomy. (Participant 7)*

#### Having a restricted life

This subcategory relates to the fact that the participants’ lives were affected by the illness; accordingly, this forced changes in their life trajectories and interpersonal interactions.

Due to repeated medical treatments, the patients’ normal study and work commitments were seriously disrupted, and thus, they lagged behind their peers to a certain extent.

*I had to take medicine every day and had to do regular follow-up checks. As a student, it seemed to take up a lot of my study time. (Participant 15)*

*Because of this disease, my work has entered a period of stasis. (Participant 14)*

Because of the limitations of the illness and the delays to study and work, the participants became out of step with others, and their social status changed. This caused the participants to spend more time alone.

*After taking a one-year leave of absence, I failed to go up to the next grade. Additionally, I lived in a rented house. I had almost no contact with my classmates. (Participant 7)*

*I was not able to socialize with others because I had severe diarrhoea and I couldn’t drink (alcohol), so I forgot the means of socialization. (Participant 4)*

Some participants perceived that they were different from others because of the illness; they assumed that others did not understand Crohn’s disease and did not want to understand patients with Crohn’s disease. The participants did not want to appear to be different from others, so they chose to isolate themselves, and thus, felt lonely.

*I didn’t have much in-depth communication with my classmates. They might not know the illness very well, and I didn’t want to make myself seem particularly different because of the illness. (Participant 7)*

Some participants thought that exposing their illness was a sign of weakness, so they did not tell others about their illness and insisted on completing their studies or work tasks even if they were suffering a relapse.

*The learning competition was extremely fierce in our college, and no one wanted to be a weak person. In this environment, even if I was ill, I told none of my classmates. (Participant 6)*

### Accepting the illness

This category relates to the fact that the participants were forced to accept that they have Crohn’s disease or were willing to accept the illness because of renewed hope. At this stage, patients’ perceptions of the illness gradually changed to “controllable and not scary”. Accepting the illness meant that the participants went out of the shadow of the illness and began to face the illness more calmly.

#### Having to accept

This subcategory reflects that participants gradually accepted that they have illness, even though they were still suffering from the illness.

After suffering from Crohn’s disease for a period of time, some participants admitted that the problems and challenges brought by the illness could not be solved by avoiding the fact that they have the illness. Thus, they had no choice but to accept the illness.

*Regardless of whether I was happy or not, the illness did not go away automatically, so I thought instead of complaining about it, it was better to face it. (Participant 12)*

In the process of accepting the illness, there was still much suffering to be resolved. Thus, participants often engaged in two strategies. One was “temporary avoidance”, which involved distracting their attention from the illness by reducing the time spent alone.

*Anyway, do not let yourself be free. When you have something to do every day, you will not think of unhappy things. (Participant 1)*

This temporary avoidance of the illness reduced the participants’ attention to the illness, but their inner embarrassment and conflict did not disappear, and the negative emotions accumulated. If the participants’ negative emotions became too difficult for them to bear, they often chose the other strategy of “discharging emotions”, such as through shopping or playing with pets, to obtain relief.

*At that time, I shopped frantically for approximately two or three months… I just wanted a way to vent. (Participant 17)*

Over time, the inner conflicts of the participants in relation to their illness still needed to be adjusted. The participants mainly used two strategies to achieve self-reconciliation and improve their acceptance of the illness. The first strategy was “lowering the standards”, which meant that the participants did not compare their current status with others but set their own standards.

*Others’ bodies scored 100 points, and my body scored 70 points. However, I can still work and take care of my family members. I have reached my own standard, and I am very satisfied. (Participant 14)*

The other strategy was “increasing the tolerance level”, which helped participants eliminate their original ideas and become more receptive. For example, some participants changed their perception of treatments such as enteral nutrition to achieve self-reconciliation.

*Eventually, my perception of enteral nutrition changed for the better because it was safe and had no side effects. If I want to eat, I can just open it (a bottle). When I thought this way, I felt more relaxed. (Participant 14)*

#### Having reasons to accept

This subcategory reflects that participants eventually felt that the “terrible and uncontrollable” illness was not as serious as they had imagined. This made the participants less fearful and gave them some hope.

When the participants discovered that Crohn’s disease is not completely uncontrollable through actively seeking or passively acquiring knowledge of the disease and the experiences of others, they became more hopeful. It is worth noting that when participants obtained comprehensive and complex illness information, the most important aspect was that they were able to screen out the positive content to gain motivation to fight the illness.

*Although it was said that the illness cannot be cured, it is not fatal. Therefore, I had hope. (Participant 14)*

The participants were also reinvigorated when they saw first-hand that patients with similar or more severe conditions could live a good life in remission.

*I shared a (ward) room with a patient. I found out later that his condition was more serious than I was previously, but he still lived his life well, which gave me confidence. (Participant 9)*

In the course of the illness, the participants found that it was normal for people to get sick, and there were often people who were less fortunate with their illness. They no longer felt they were the only ones suffering the illness or the most unlucky.

*I noticed there were many patients with Crohn’s disease, and many of them were very young. Therefore, it was acceptable, and I was not the unluckiest person. (Participant 18)*

After learning more about other types of illnesses, the participants realized that Crohn’s disease is no more special than any other chronic illness, such as diabetes or hypertension.

*I thought it was similar to hypertension, which is also lifelong and requires long-term medication. Crohn’s disease is not special. (Participant 15)*

The participants’ personal experiences of remission also helped reduce their fear of the illness. With their increasing hope, they cooperated more actively with the treatment. As the symptoms were alleviated, the psychological state of the participants also improved, thus forming a virtuous circle.

*Because the treatments were effective, I followed the doctor’s advice and controlled my diet very well. Then, my mental state improved, and the symptoms gradually decreased. (Participant 6)*

The participants also found that they were not alone in fighting the illness. Other people provided much support to them, which created a strong foundation. For example, family care and financial support gave participants more confidence to adhere to the treatment and overcome the illness. The professional medical team was also a strong force of support for them.

*I’m very grateful to my parents who have been behind me and have given me a lot of support. They tried their best to relieve my pain. (Participant 9)*

*The doctors were very attentive to me. There were also nice nurses who helped me change the dressing very gently and told me that listening to music can relieve the pain. (Participant 15)*

### Dancing with the illness

This subcategory reflects that participants interacted with the illness day in day out, which gradually improved their ability to cope with relapse and maintain remission. The Crohn’s disease is a lifelong affliction, and thus, the participants began to realise that they needed to find ways to reconcile their life founded on illness. The self was reconstructed by dancing with the illness, and the participants became more positive and resilient compared to the previous stage.

#### Responding according to the situation

This subcategory reflects that participants made adjustments and changes to enhance their ability to coexist with the illness based on the current problems they were experiencing.

To better cope with relapse, the participants took the initiative to obtain knowledge and experience from various channels.

*Since I obtained certain knowledge of the illness, I know when I can deal with it by myself and when I must go to a doctor for help. (Participant 18)*

The participants also learned to explore lifestyle changes to better adapt to a life with the illness.

*When I go out, I must know the location of the bathroom in advance. This can avoid some embarrassing situations. (Participant 4)*

During a relapse, some participants were unable to solve problems by themselves, so they turned to the people around them for help.

*I told my friends and leaders that I had a gastrointestinal illness and that I cannot eat spicy food or drink (alcohol). I said that I hoped they could take care of me in terms of diet. (Participant 11)*

Apart from coping with relapse, maintaining remission was also an important goal for the participants. Self-reflection was important for the participants to understand the significance of maintaining remission through self-management. Self-reflection mainly included three aspects. The first aspect was “perceiving individual responsibility”, where participants realized that their remission could reduce the burdens on their families.

*I did not want my family to worry about me. I changed myself step by step. (Participant 12)*

The second aspect was “perceiving harms from not changing”, which refers to the fact that when the participants saw the severe condition of other patients after exacerbation of the illness or when they experienced their own relapse due to not paying attention to the illness, they realized the importance of self-discipline.

*I once completely disregarded my diet, which caused my body to have a big reaction the next time I took infliximab. My ex-girlfriend was shocked when seeing my suffering from the illness, which led to a breakup. So, I thought some positive changes must be made. (Participant 7)*

The third aspect was “perceiving positive outcomes after changing”, where participants learned that other patients had achieved better results in alleviating the condition through self-management, so they also tried it.

*Fellow patients were a good example. I thought that if I was able to learn a lot about the illness like they did, I could also live relatively steadily and normally. So, I followed them. (Participant 18)*

After self-reflection, the participants began to engage in self-discipline in many aspects of their lives and persisted in doing so.

*I control my diet, and I engage in proper exercise. I always do Tai Chi… Regular check-up is also very important. (Participant 16)*

With great experience coping with relapse and maintaining remission, the participants gradually took control of their lives with the illness, and changes in the status of the illness no longer had significant impacts on the patients’ moods and daily lives.

#### Planning for future

This subcategory reflects that the participants made plans and took actions to follow these plans by imagining possible bad situations that could occur in the future and the goals they wanted to achieve under such situations.

The illness experience made the participants realize that the future is uncertain and that they need to be prepared for the worst. For example, some participants imagined a situation in which they were unable to pay their medical bills.

*If I become poor, I will try to visit a doctor of traditional Chinese medicine. I will definitely try to work, even if I’m a waiter in a small hotel. (Participant 5)*

The worst outcome that some participants planned for was death. They chose to communicate their plans regarding death with their families to avoid causing themselves and their families to feel regret.

*I assumed the worst. If I am facing death, what should I do? I communicated with my parents and made it clear what to do when that time comes. (Participant 17)*

The sense of uncertainty made the participants realise that they had a responsibility to secure the quality of their future life. They realized that other people cannot offer economic and medical support to them forever and that everyone should depend on themselves.

*After getting sick and divorced, I found I had to rely on myself… If you rely on yourself at first, you will not care about divorce, and you will still be able to live. (Participant 9)*

When the participants made efforts to secure their future lives, they were often most concerned about economic pressures. In planning for the future, many participants put goals such as increasing their income and securing a stable job as priorities.

*At present, I still want to make enough money in the short term to pay for my medical costs. It is always my priority. (Participant 7)*

The participants also valued health care. They planned to live in provinces with better medical benefits and conditions. To have such choices in the future, they were motivated to continue their current study and work.

*After getting sick, I have been longing to go to a place with good health benefits and high medical service quality. I became more determined to study hard and had a clearer goal for the future. (Participant 15)*

### Enriching life beyond the illness

This category reflects that participants began to try new things in other areas of their lives to enrich their life experiences and, to a certain extent, this allowed them to obtain self-satisfaction and realize their self-worth. At this stage, the participants were able to get along well with the illness; they were even able to break through the limits of the illness. Meanwhile, the illness became less and less threatening to the self, until it only became a part of the self.

#### Exploring the joys of life

This subcategory reflects that the participants explored and engaged in opportunities that broadened their horizons and cultivated their tastes.

After suffering from the illness, the participants felt the uncertainty and impermanency of life, and thus, they appreciated life more. They wanted to feel less regret by enriching their life experiences.

*I tried some sports that I have never done before, such as mountain climbing. It was delayed by the illness before, so I would like to try more things when I am well. (Participant 3)*

Some participants devoted more time to the things that they enjoyed. They developed hobbies based on their own preferences.

*Now I’m teaching myself the ukulele… I always wanted to learn a musical instrument, but I could not find time. Now I have a lot of time to do it. (Participant 1)*

Some participants combined personal hobbies with actual needs, hoping to make some contribution to society.

*If I learn Japanese well, I can help our inflammatory bowel disease foundation when we go abroad for exchanges. Meanwhile, I like watching anime, which also led me to learn Japanese. (Participant 19)*

Some participants developed new interests because of the illness. For example, they discovered their love for biomedical knowledge.

*During the course of my illness, I read a lot of English papers… I have studied this illness to an extent, and I am quite interested in it… So, I plan to learn more in this area in the future. (Participant 6)*

#### Expanding the meaning of life

This subcategory reflects that, to realize their self-worth, the participants returned to work, helped others or explored new ways to achieve breakthroughs.

After the condition stabilized, some participants were not satisfied with their status and tried to achieve changes. They returned to their previous posts, wanting to improve their level of professionalism.

*I try my best to do my present job well, which gives me a great sense of achievement. (Participant 14)*

Either because they wanted to give back by providing the help they received or because they wanted to help patients avoid detours, many participants chose to help people in need.

*My desire is to help those children who have the same experience as me. I hope to help them find the right direction instead of taking a detour like me for many years. (Participant 3)*

For many participants, helping others helped them realize their self-worth and this was a manifestation of the meaning of life.

*As a volunteer, some patients contact me. If my communication can help them to face life and illness more bravely, I will feel my life is still valuable. (Participant 14)*

Some participants wanted to resume challenging plans that they were unable to carry out because of the illness.

*I want to make up for the previous plans that were left behind, like travelling to Tibet. (Participant 12)*

## Discussion

A theoretical understanding of the reconstruction of the self after the diagnosis of Crohn’s disease was developed in this study. The patients constructed a new self during the four stages of growth, but this self-reconstruction was not a steady forward process. The self may regress to the past due to new pains experienced or may stay at a stage for a long period of time before moving forward. However, as a patient’s ability to cope with the illness becomes stronger, the self becomes relatively stable. This has also been demonstrated in the identity reconstruction of patients with chronic fatigue syndrome [40]. Compared with Tedeschi and Calhoun’s posttraumatic growth model [19, 21] on acute seismic trauma, which usually lasts for a limited time period, the current study focused on a chronic trauma that fluctuates and persists for a lifetime. Moreover, Tedeschi and Calhoun’s model [19, 21] was characterized by a linear cognitive process with many influences. However, the current study revealed that the process is a reversible cycle, with an overall trend of growth. Although there are some similarities between our finding and Zhai’s [25], such as viewing posttraumatic growth as a fluctuating cognitive and emotional adjusting process of living with illness, our finding is different as we interpreted the posttraumatic growth process from the perspective of reconstructing self through interacting with the disease.

The reconstruction of the patient’s self has its own characteristics at each stage of growth. At the stage of “suffering from the illness”, the illness poses a great threat to patients. Most patients view the illness as uncontrollable and terrible and are prone to experiencing serious negative emotions, such as fear, helplessness and self-pity. According to the assumptive world theory, individuals hold core assumptions about the world and the self; these assumptions help individuals to understand their own experiences and create a sense of order and stability [41, 42]. The diagnosis of Crohn’s disease was a trauma that shattered patients’ previous core assumptions. The conflict between the real world and the hypothetical world causes psychological stress, leading to a meaningless state. This phenomenon has also been reported in studies of multiple sclerosis and systemic lupus erythematosus patients [43, 44].

As the illness progresses, the life routine of the patient is disrupted. Similar to previous studies [45], patients with Crohn’s disease often reported self-denial and felt that they were different from others and that others could not understand their suffering, which led to self-isolation. Self-isolation has also been observed in the posttraumatic growth of Chinese patients with spinal cord injury [46] and breast cancer [25]. In Chinese culture, “sickness” and “suffering” are Chinese characters that are synonymous with “shame” [47], which means that being diagnosed with an illness is a stigmatizing event. Meanwhile, the Chinese public has a low awareness of Chron’s disease, this increases the risk of stigma [48]. In our study, some participants thought that exposing their illness was a sign of weakness and they did not want to appear to be different from others. Self-isolation was particularly pronounced in the early stages of the illness. At this stage, the illness posed a great threat to patients and this threat was, to some extent, the engine of posttraumatic growth [49], which prompted the patient to reflect, learn and embark on the road of growth.

“Accepting the illness” is a sign that patients with Crohn’s disease are beginning to grow. Similar findings have been observed in accidentally injured patients [50]. When a patient said, “I have no choice but to accept it”, this indicated that the patient had begun to accept the illness. At this stage, patients are eager to change and easily accept external support. This is a good time for healthcare professionals to carry out psychological interventions to promote the growth of the patients. “Accepting the illness” can be explained as a process from challenged core assumptions to re-establishing the assumptive world. In the current study, several strategies were adopted for this purpose, such as, comparing one’s self with other patients, diseases or events. This promoted the transformation of self-positioning from “victim” to “survivor” and led to the re-examination of inherent concepts and the patient’s original life. This comparison method has been described in previous studies of patients with Crohn’s disease [27, 51], indicating that the application of this method has a certain universality and effectiveness. When patients said, “I’m not the unluckiest”, and had fewer complaints about the illness, it demonstrated improved acceptance of the illness with the achievement of self-reconciliation, to a certain extent.

Some patients with Crohn’s disease entered the “dancing with the illness” stage when they realized that their own strength is the key factor in coping with relapse and maintaining remission. The participants became more positive and resilient compared to the previous stage. Effective self-reflection, including “perceiving individual responsibility”, “perceiving harms from not changing” and “perceiving positive outcomes after changing”, promoted the growth of patients with Crohn’s disease. Healthcare professionals can actively explore the self-management potential of patients from these three aspects. Culturally, China is deeply influenced by Confucianism [28], which views a person as part of a family with interdependent responsibilities and expectations [52]. Hence, " responsibility” is closely tied to the family. For example, participants realized that remission of their disease could reduce the burden on their family and thus, they were motivated to grow to fulfil their family obligations. This responsibility within the family culture contributes to a higher level of growth.

Crohn’s disease is a lifelong fellow traveller, which means that patients may experience a shattering of “assumptions” more than once. Therefore, “dancing with the illness” emphasizes the reconciliation of the self with the illness, rather than leaving the self and the disease alone or blindly controlling the illness. This can be explained by the philosophy of Yin and Yang in the Tai Chi diagram. Yin-Yang and Taiji are the basic concepts of Zhouyi (a famous early philosophical work in China) which expresses the basic views of Chinese people on the universe and life. Taiji is the noumenon of the universe, and Yin and Yang respectively represent the static and dynamic states of Taiji. When Yin and Yang balance, Taiji becomes a balanced whole [53]. The self and illness seem to be contradictory, yet in the Chinese cultural background, patients are more inclined to achieve growth among contradictions. Research suggests that Westerners and Easterners view stability and change over time quite differently. Westerners living in independent cultures value a consistent self and see private attributes as self-defining, while Easterners within interdependent cultures view change and contradiction as essential to the makeup of a complex self [32, 54, 55]. Thus, faced with a traumatic event, Chinese people may be inclined to adjust the self to the situation they encounter and achieve growth based on reconciliation and coexistence. These conceptualizations of the self and crisis situations provide a nuanced understanding of posttraumatic growth from the perspective of culture.

In addition, patients with Crohn’s disease often planned for the future to increase their hope and confidence. There are two explanations for this finding. First, according to image theory [56], the imagination of possible scenarios in the future provides the basis for decision-making regarding future plans, and this imagination is mostly based on the present. Second, according to socioemotional selectivity theory [57], when individuals perceive that they have enough future time, they pay more attention to future-oriented goals; that is, the acquisition of knowledge to acquire information and learn social skills, rather than the regulation of emotions, which focuses on meaning and emotional intimacy. When patients with Crohn’s disease entered the stage of “dancing with the illness”, the perceived threat of the illness was reduced. Moreover, since young and middle-aged patients are in the prime of their life, they often realise that there is enough time in the future, so they tend to make future plans related to learning social skills and other aspects.

When the patients returned to normal life and accepted the illness as a part of themselves, the self was no longer bound by the illness; instead, they had the power to break through the limitations of the illness and were constantly seeking an “enriched life beyond the illness”. On the one hand, patients “explored the joy of life” by trying more things or developing their own hobbies. A study by Kampman et al. [58] also found that severely injured patients developed new personal skills as a result of managing their disabilities. According to Joseph and Linley [59], the struggle against trauma enables individuals to understand and modify their own goals, values and interests to bring them closer to their real inner selves. On the other hand, some patients with Crohn’s disease created new life goals through the pursuit of life meaning, replacing the goals and beliefs that they had shelved due to the illness. Studies have shown that in the face of adversity and trauma, the recreation of meaning plays an important role in maintaining mental health and helps patients continue to live and grow [60, 61]. Therefore, the creation of life meaning has a certain universality and special significance in posttraumatic growth. At this stage, clinical staff can help patients recall and reflect on their life goals before the illness and the value of the life they desire to achieve. This can prompt patients to rethink and re-examine their lives and explore and discover their potential meaning of life.

## Conclusion

This study involved in-depth interviews with 19 patients with Crohn’s disease to develop an interpretive understanding of posttraumatic growth. The core category identified was “reconstructing self from the illness”. The findings revealed that the illness brings a double blow to the patient’s body and mind, placing restrictions on the lives of patients. Driven by pain, patients rely on their own efforts and external support to reflect, learn and constantly adjust their inherent cognition and their self to achieve growth. According to the characteristics of growth identified here, healthcare providers can carry out targeted psychological interventions to improve a patient’s level of posttraumatic growth and quality of life. In the future, more research is needed in the area of posttraumatic growth in order to develop psychological interventions to improve patient outcomes.

## Limitations

This study has some limitations that should be noted. First, the participants had relatively high education levels, and the participants were all in remission or experiencing mild disease; thus, these participants may not represent the general Crohn’s disease population. Second, video and telephone interviews were adopted in some cases due to the impact of COVID-19. This had an impact on the acquisition of nonverbal and environmental information during the interviews.

## Data Availability

The data generated during and/or analysed during the current study are not publicly available due to ethical issues but are available from the corresponding author or first authors on reasonable request.
